# Type D Personality and Stomatognathic System Disorders in Physiotherapy Students during the COVID-19 Pandemic

**DOI:** 10.3390/jcm10214892

**Published:** 2021-10-23

**Authors:** Magdalena Gębska, Bartosz Dalewski, Łukasz Pałka, Łukasz Kołodziej, Ewa Sobolewska

**Affiliations:** 1 Department of Rehabilitation Musculoskeletal System, Pomeranian Medical University, 70-204 Szczecin, Poland; mgebska@pum.edu.pl (M.G.); lukas@hot.pl (Ł.K.); 2 Department of Dental Prosthetics, Pomeranian Medical University, 70-204 Szczecin, Poland; bartosz.dalewski@pum.edu.pl (B.D.); rpsobolewski@wp.pl (E.S.); 3 Private Dental Practice, Rzeszowska 2, 68-200 Żary, Poland

**Keywords:** COVID-19, stomatognathic system, type of personality, type D personality, TMD, orofacial pain, masticatory

## Abstract

Background: A person’s response to stressors is largely dependent on their personality traits that affect the way stress is controlled and relieved. This article is a quantitative analysis assessing the importance of the distressed personality in the development of stomatognathic system disorders (SSDs) in physiotherapy students during the COVID-19 pandemic. Objective: The goal of the research was to assess the presence of type D personality in students with symptoms of stomatognathic system disorders. Material and Method: The research was carried out among 300 physiotherapy students. The data were collected using the form of the occurrence of symptoms of SS disorders developed for the purpose of the study and the standardized psychological DS14 questionnaire. Results: In a group of 300 students, the presence of type D personality was found in 160 people (53.3%). People with type D personality had symptoms of SS disorders more often than the group without stressful personality traits. There was a significant difference between the groups regarding all the examined symptoms. In the group of people with type D personality, the most frequently reported symptoms of SS disorders included: headache (51.3%), pain in the neck and shoulder girdle (43.1%), and teeth clenching (35.6%). As many as 70% of the respondents in the group with symptoms of SS disorders (P1) had type D personality, whereas in the asymptomatic group (P2) this result was 23.3%. There was a significant difference between the groups (*p* = 0.00). Statistically significantly higher values of both D personality dimensions were observed in women than in men with symptoms of SS disorders. In people reporting symptoms of SS disorders, higher average values were observed in both dimensions of type D personality. There were significant differences between the groups. Conclusion: type D personality may contribute to the development of symptoms of stomatognathic disorders.

## 1. Introduction

The COVID-19 pandemic disrupted the education system in Poland and vastly contributed to social isolation, with all due consequences. That, in turn, had a negative impact on the mental and physical health of students and university graduates. The accompanying stressors could have had influenced the development of increased masticatory muscles tension and numerous parafunctions, and thus the formation of stomatognathic system dysfunctions [1,2,3,4,5]. The relationship between increased stress and disorders of the stomatognathic system and quality of life has been widely described in the literature [6,7,8]. For example, bruxism, the diurnal and/or nocturnal unconscious teeth clenching and grinding, is commonly triggered by environmental stressors and depends on the personality traits that affect the way stress is controlled and relieved [9,10,11].

Therefore, the authors of the present study aimed to evaluate the relationship between the occurrence of symptoms of SS disorders and type D personality in physiotherapy students during the COVID-19 pandemic. 

Regarding health psychology, there are three personality types that favor the development of somatic diseases: type A (‘coronary personality’), type C (‘cancer-prone personality’), and type D (‘distressed personality’) [12]. Two approaches are taken into account regarding the study of the relationship between personality and disease. The first indicates that certain personality traits are associated with morbidity rate of certain severe health issues such as, for example, type A personality promotes ischemic heart disease, or type C, which was previously associated with cancer [12,13,14]. The second approach assumes the existence of a general susceptibility to disease and indicates that this susceptibility is the result of personality traits that may favor or inhibit the development of the disease [15]. The growing interest in the problem of the relationship between personality and somatic diseases took place at the end of the 1990s, when Johan Denolett introduced the concept of distressed personality into the literature (distressed personality), i.e., type D personality, which he emphasized at the early 2000s [16,17].

Type D consists of two main dimensions, treated as relatively constant personality traits, i.e., negative affectivity (NA) and social inhibition (SI). Negative affectivity is expressed as a tendency to experience strong negative emotions, such as anxiety, anger, irritation, and hostility. On the other hand, social inhibition is associated with the tendency to refrain from expressing negative emotions and behavior consistent with these emotions. Refraining from revealing emotions is conscious and is undertaken mainly in social situations, primarily for fear of disapproval and rejection by other people [18]. People with a type D personality tend to worry, they feel tense and blame themselves which may result in suicidal behavior [19]. They are pessimistic about the world, have low self-esteem and a low level of life satisfaction. Moreover, they show weak bonds with other people, and use sedatives more frequently [20,21]. 

Type D personality shows some similarities to other personality characteristics that contribute to the development of somatic diseases, such type A or type C, but above all, to the two dimensions of personality that make up the ‘Big Five’, i.e., neuroticism and introversion. This relationship was confirmed by De Fruyt and Denollet [22].

The theoretical foundations of the type D personality refer to the biological theory of inhibition and activation formulated by Eysenck [23]. According to this theory, people for whom the stimulating potential is created quickly and with great force and the reactive inhibition appears slowly and disappears quickly, show a tendency towards introverted behavior (for extroverts it is the other way around). On the other hand, the theory of activation refers to individual differences in the level of activity of the cortico-reticular loop that determines the level of activation. The activation level of introverts exceeds the level of activity of extroverts [24]. The tendency to experience negative emotions, characteristic for both type D personality and neuroticism, modifies the behavior and level of functioning in situations requiring the activity of the limbic system [25]. 

Type D personality is linked to neuroticism by a tendency towards a catastrophic perception of reality, a way of evaluating events as highly threatening and harmful and a feeling of strong anxiety and tension [12]. What is characteristic in social situations is confusion, shyness in the presence of others, a tendency to worry, a pessimistic view of the world, high susceptibility to stress, and a tendency to break down in difficult situations. They are differentiated by the fact that in the type D personality, there is an emphasis on refraining from revealing negative emotions. It is also combined with introversion by refraining from keeping social contacts and shyness [14]. Moreover, introversion, similar to type D, is associated with a lower tendency to search for social support, poorer quality of social contacts and low self-esteem [26]. Therefore, it can be assumed that type D personality is the equivalent of neurotic introversion, yet people with type D personality would feel stress more strongly and experience its’ consequences that appear to be more robust for physical and mental health. 

The aim of this study was to evaluate the presence of type D personality in students with symptoms of stomatognathic system disorders. We hypothesized that type D personality traits may contribute to the development of symptoms of stomatognathic dysfunction during the COVID-19 pandemic.

## 2. Materials and Methods

The research was carried out from October 2020 to June 2021 among 300 students of physiotherapy at the Pomeranian Medical University in Szczecin. The study group (P1) consisted of 150 students who reported symptoms of SSDs in the questionnaire. The control group (P2) had the same number of participants without SS symptoms.

Inclusion criteria in the control group were: first, second, or third year students of physiotherapy studying in a hybrid system (stationary and remotely); no symptoms of SS disorders reported in the past; age 20 to 35; consent to participate in the study. Exclusion criteria were: chronic diseases, including psychosomatic diseases; during or after treatment of SS disorders, and pregnancy. The differentiating factor in the inclusion criteria in the P1 and P2 groups was the presence of symptoms of SSDs in the P1 group. 

### 2.1. Research Tools

The SSD disorder assessment questionnaire consisted of 10 closed questions concerning the occurrence of symptoms related to the temporomandibular joints, masticatory muscles, and the cervical spine. A standardized DS-14 psychological questionnaire was carried out among all students (Type-D scale). The questionnaire consists of seven questions which concern the tendency to experience various negative emotions and seven concerning tendencies to refrain from expressing these emotions. To qualify as Type D, you must obtain a minimum of 10 points in each of the two dimensions i.e., NA and SI.

### 2.2. Characteristics of the Study Group

A total of 194 women (64.7%) and 106 men (35.3%) participated in the study. Among all the respondents, there were 30.3% of first-year students, 36% of second-year students and 33.7% of third-year students. The average age of the respondents was 21.71 (SD 2.69) and 22.26 (SD 2.10), *p* = 0.05, in the P1 and P2 group, respectively. The group of students with symptoms of SSDs included 103 women (68.7%) and 47 men (31.3%), and the group of asymptomatic participants consisted of 91 women (60.7%) and 59 men (39.3%). There was no difference in the gender structure between the groups, *p* = 0.14723. 

After analyzing the data from the SS disorders symptom questionnaire in the P1 group, it was found that the most frequently reported symptoms were: headache 68%, pain in the neck and shoulder girdle 58%, teeth clenching 50%, TMJ acoustic symptoms 33.3%, TMJ pain 32.7%, increased masticatory muscle tension 30%, teeth grinding 22.7%, facial pain 12%.

The statistical comparison of the results of the SS disorder questionnaire between the groups was omitted due to the lack of occurrence of the studied variables in the P2 group.

### 2.3. Statistical Analysis

Data are presented as n and % of responses for qualitative variables and the average +/− standard deviation for quantitative features. The Chi 2 Pearson test was used to compare the relationships between the qualitative variables. Comparisons for quantitative variables were made using the Student’s *t*-test, the relationship between quantitative variables was assessed using the Pearson correlation coefficient. Due to the large number of cases in the study groups, parametric tests were used based on the central limit theorem. The analysis was performed using the Rstudio package (RStudio, PBC) and *p* values < 0.05 were considered significant.

## 3. Results

According to the obtained data the presence of type D personality was confirmed in 160 participants (53.3%). The results of the assessment of the presence of type D personality in the P1 and P2 groups are presented below (Table 1).

As it can be seen from Table 1 as many as 70% of the respondents in the group with symptoms of SSDs (P2) had type D personality, whereas in the asymptomatic group (P1) this result was 23.3%. There was a significant difference between the groups (*p* = 0.000). 

Comparing the occurrence of symptoms of SS disorders between people with and without type D personality, the following results were obtained (Table 2). 

The results presented in Table 2 show that people with type D personality experienced symptoms of SS disorders more frequently than in the group without distressed personality traits. There was a significant difference between the groups regarding all the examined symptoms. In the group of people with D personality, the most frequently reported symptoms of SSDs included: headache, pain in the neck and shoulder girdle, and teeth clenching.

As shown in Table 3, significantly higher values of both type D personality components were observed in women than in men with symptoms of SS disorders. 

**Figure 1:**
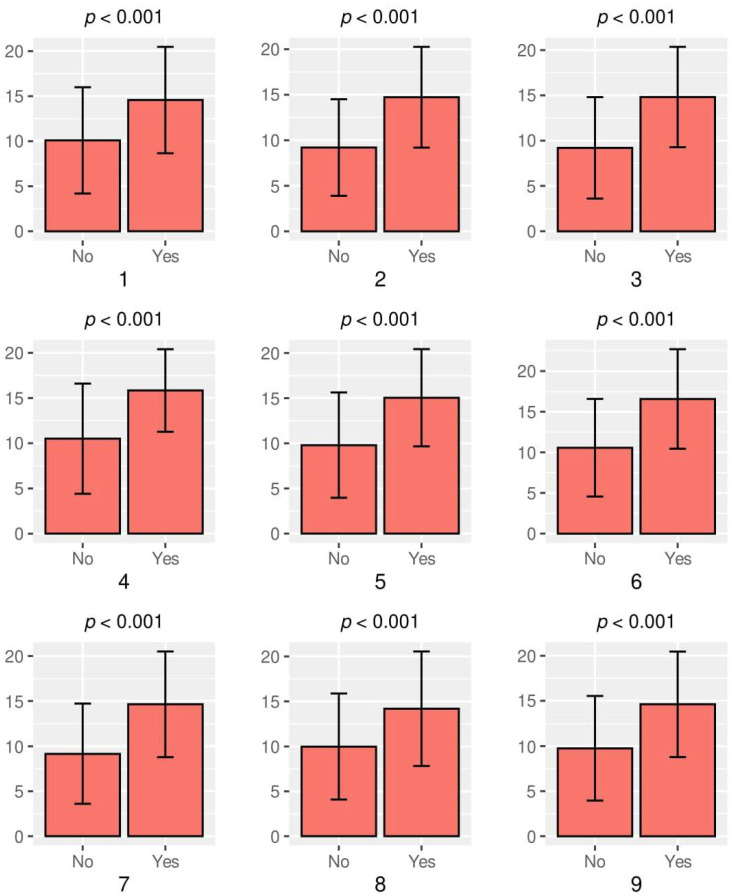
Analysis of the negative affectivity (NA) in relation to the symptoms of SSDs. Legend: 1—TMJ Pain, 2—Headache, 3—Pain in the neck and shoulder girdle, 4—Facial pain, 5—TMJ acoustic symptoms, 6—TMJ blocking, 7—Teeth clenching, 8—Teeth grinding, 9—Increased masticatory muscles tension.

**Figure 2:**
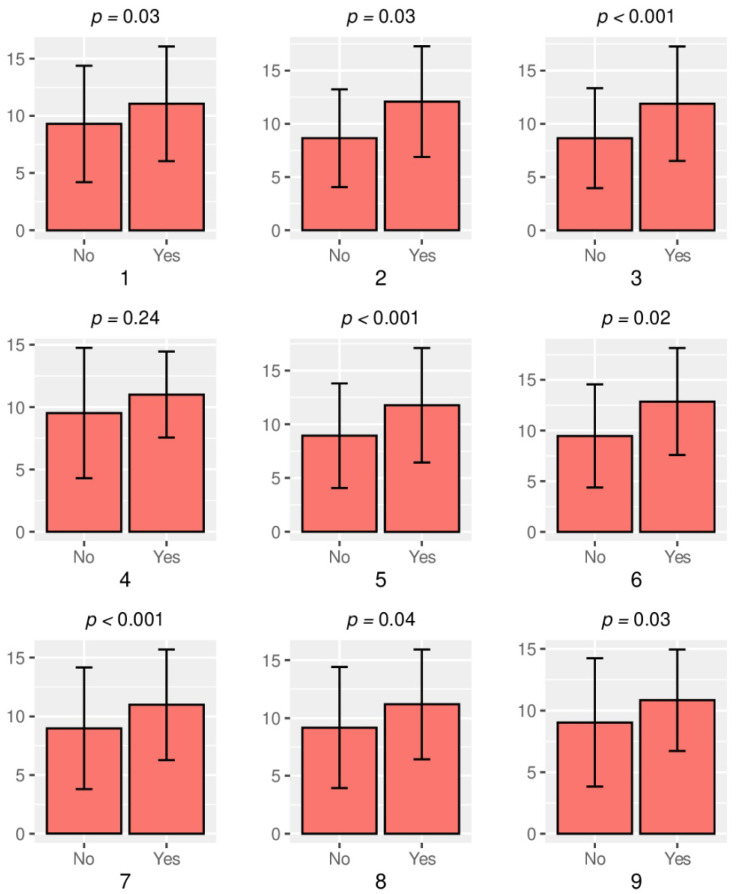
Analysis of the social inhibition (SI) in relation to the symptoms of SSDs. Legend: 1—TMJ Pain, 2—Headache, 3—Pain in the neck and shoulder girdle, 4—Facial pain, 5—TMJ acoustic symptoms, 6—TMJ blocking, 7—Teeth clenching, 8—Teeth grinding, 9—Increased masticatory muscles tension.

Below in Figure 1 and Figure 2 the analysis of both dimensions of type D personality in relation to symptoms of SSDs is presented. 

Figure 1 and Figure 2 present the results obtained after the analysis of NA and SI occurrence in relation to the reported symptoms of SSDs. In people reporting symptoms of SS disorders, higher average values were observed in both dimensions that make up the type D personality (NA and SI). There was a significant difference between people with and without symptoms. In the symptomatic group, the average NA scores are higher than SI. 

## 4. Discussion

According to the present research carried out on a group of 150 physiotherapy students, the most common symptoms of SS disorders were headaches (51.3%), neck and shoulder girdle pain (43.1%), and teeth clenching (35.6%). The studies carried out by Glaros et al. show that involuntary tooth contact is one of the most typical parafunctions observed more frequently in TMD (temporomandibular joint dysfunction) patients than in healthy people [27]. Similar conclusions were also drawn by Moreno et al., who found a correlation between TMJ disorders and head and neck pain [28]. According to Emodi-Perlman et al., the COVID-19 pandemic had a significant negative influence on the psycho-emotional state of the Israeli and Polish populations, leading to the worsening of bruxism and TMD symptoms [29]. De Medeiros R.A. et al. drew similar conclusions by conducting research on Brazilian medicine students. According to their study, social isolation and stressful situations caused by the pandemic may increase the number of people with TMD symptoms [30]. For several years, great interest of researchers and practitioners has been aroused by the so-called ‘distressed personality’—type D. It is considered a risk factor for somatic diseases and is of high importance as far as the perception of the environmental stress is concerned [31].

In the author’s own research on a group of 300 physiotherapy students, the presence of type D personality was found in 160 participants (53.3%). In accordance with O’Riordan et al., type D people perceive their life events as significantly more stressful than people with a different personality type. Type D personality people also report increased perceptions of negative social relationships and less social support [20]. What is more, people with type D personality are characterized by a worse quality of life and they assess their own health condition as worse [32]. In the context of the studied group- future physiotherapists- numerous studies should also be cited, in which it was shown that employees characterized by type D personality perceive their work environment as more stressful with more frequent occurrence of occupational burnout syndrome [33,34,35,36]. According to the authors, the above-mentioned considerations indicate that the high prevalence of type D personality among physiotherapy students may become an obstacle in providing effective rehabilitation services to patients.

The results of many studies have shown that the type D personality is a significant predictor of cardiovascular diseases (ischemic heart disease, hypertension), as well as somatic diseases, such as cancer, peptic ulcer disease, and skin diseases [37,38,39,40,41].

In a study by Condén et al., a strong relationship was found between the type D personality with psychosomatic symptoms and musculoskeletal pain [42]. 

However, there are no scientific studies on the evaluation of the relationship between the type D personality and the occurrence of symptoms of stomatognathic system disorders. The studies presented in the literature, taking into consideration related constructs, especially neuroticism, confirm the role of the experience of negative emotions and reserves in relations with other people as those elements of personality that favor the assessment of stress and intensify its the negative effects. According to Moayedi et al., neuroticism may contribute to the pathophysiology of muscular TMD because there is a correlation between chronic pain in TMD and the patient’s neurotic personality [43]. 

On the other hand, according to the research of Southwell et al., TMJ dysfunctional individuals obtain higher results regarding neurotic and introversion scales [44]. Serra-Negra et al. have shown that children with a high level of neuroticism in their personality domain are more prone to sleep bruxism [45]. 

Research over the past twenty years has indicated a connection between several psychological variables and TMD [46,47,48]. Indeed, it is easy to observe differences in the severity of personality traits, experienced levels of stress, depression, and catastrophic situations between TMD patients and those without the disorder. A well-known example of the association of psychosocial risk factors in chronic TMD is the insecurity that accompanies long-term suffering [48]. What is more, it should also be noticed that there are psychological factors associated with the initial pain symptoms in TMD, as well as socio-demographic variables that contribute to the perception of craniofacial pain [48]. One of the most important determinants influencing the behavior and functioning of an individual is their personality.

The authors of the present study, by utilizing the DS-14 questionnaire, which is used to evaluate the occurrence of type D personality, have observed that it is significantly more common in people reporting symptoms of SS disorders than in asymptomatic people. (*p* = 0.00000). These results suggest that people with symptoms of SS disorders have personality traits that make them prone to perceived life stress. It is also worth mentioning that significantly higher values of both type D personality components (NA and SI) were observed in women than in men with symptoms of SS disorders. The above-mentioned data may prove that not only type D personality may predispose to SS disorders, but also gender may be an additional factor influencing the frequency of SS dysfunctions. Yeow’s research on a group of 157 psychology students has shown that 50% of men and 46.4% of women had a type D personality. Moreover, women had higher HS when compared to men [49]. Cho S. et al. have observed that type D personality is related to the level of perceived stress. Additionally, it was concluded that there are gender differences in type D personality, stress, and stress coping strategies [50]. 

The authors of the present article, by comparing both dimensions of the type D personality (NA and SI), noted statistically significant differences between the groups in terms of symptoms, i.e., headache, face pain, TMJ and shoulder girdle, acoustic symptoms and TMJ blocking, teeth clenching and grinding as well as increased masticatory muscles tension. In the symptomatic group, the NA dimension showed higher average values than SI, that is related to the individual’s tendency to experience strong negative emotions such as anxiety, anger, irritation, and hostility. After analyzing the obtained data, it can be claimed that the type D personality, and above all the dimension of negative affectivity, is one of the predictors of symptoms of SS disorders. The research results presented in the literature indicate a relation between the NA dimension and unfavorable eating and shopping habits, symptoms of depression, anxiety, insomnia, alcohol abuse and a low level of life satisfaction. 

There are many strategies and methods in the field of psychotherapeutic treatment applied to individuals with a disturbed personality. Currently, the main method is a long-term psychodynamic psychotherapy. The literature offers numerous reliable studies confirming the effectiveness and usefulness of this treatment procedure if conducted by a psychologist [51,52,53]. If the disorder is correctly qualified and the therapy is durable (long-term in nature), it is possible to obtain real changes in the patient’s functioning, which are conducive to better functioning in many aspects of their life. One can also find a growing number of reports confirming the effectiveness of other methods of psychotherapy (e.g., modifications of cognitive-behavioral therapy) [54]. It cannot be forgotten that the basic therapeutic factor, independent of the applied psychotherapeutic approach, is establishing a therapeutic relationship between the therapist and the patient, based on mutual trust, safety, and commitment. The progress in the field of psychotherapy of personality disorders observed in recent years is a hope for many patients, who can thus avoid gradual degradation of their lives and have a chance to function better on daily basis. Pharmacotherapy as a method of treating personality disorders is of complementary importance and psychotropic drugs are recommended mainly for symptomatic indications, e.g., to control severe depression, anxiety, or short-term psychotic symptoms [55].

By analyzing the available scientific literature and the results of our own research, it is possible to determine with high probability that the distressed personality may have a great influence on the functioning of the stomatognathic system, in particular on the development of the masticatory organ parafunctions and head and neck pain.

Further research is necessary to better understand the impact of the type D personality on the occurrence of stomatognathic system disorders.

## 5. Limitations

The presented results, indicating the predictive role of the type D personality in the development of symptoms of SS disorders, should be treated with great caution. First of all, due to the cross-sectional nature of the research and also due to the small group of respondents. One should also remember about the multifactorial determinant of SS disorders, which means that personality is only one of the numerous factors determining the occurrence of SSDs. A particularly important limitation of the presented studies is the lack of assessment of occlusal conditions, including the occurrence of malocclusion in the study group, which constitutes an important etiological factor of the stomatognathic system disorders. In addition, possible missing teeth and traumatic nodes within the masticatory apparatus may be the cause of the appearance of micro and macro injuries in the examined system. Therefore, the authors see the need to continue the research carried out, taking into account the role of occlusion in the formation of SSD.

## 6. Conclusions

1. In students with a distressed personality, psychological programs should be implemented, including education and training courses for future graduates, so that they can effectively deal with negative affectivity and social inhibition.
2. Personality type assessment should become one of a mainstays in TMD evaluation, as well as personalized psychological coping strategies an essential part of management.
3. People with type D personality and symptoms of SS disorders should receive psychological support, focused directly on intricate connections between possible somatization issues and catastrophizing aspects of this complex personality trait.

## Figures and Tables

**Table 1:** The occurrence of type D personality in groups P1 and P2.

Variable	Group P1	Group P2	Statistical Significance
Type D personality	115 (76.7%)	45 (30%)	*p* = 0.000
No Type D personality	35 (23.3%)	105 (70%)	

**Table 2:** The occurrence of symptoms of SS disorders in students with and without type D personality.

Variable		No Type D Personality	Type D Personality	Statistical Significance
TMJ Pain	no	128 (91.4%)	122 (76.3%)	*p* = 0.001
	yes	12 (8.6%)	37 (23.1%)	
	I don’t know	0 (0%)	1 (0.6%)	
Headache	no	120 (85.7%)	77 (48.1%)	*p* = 0.000
	yes	20 (14.3%)	82 (51.3%)	
	I don’t know	0 (0%)	1 (0.6%)	
Pain in the neck and shoulder girdle	no	122 (87.1%)	90 (56.3%)	*p* = 0.000
	yes	18 (12.9%)	69 (43.1%)	
	I don’t know	0 (0%)	1 (0.6%)	
Facial pain	no	138 (98.6%)	142 (88.8%)	*p* = 0.002
	yes	2 (1.4%)	16 (10%)	
	I don’t know	0 (0%)	2 (1.3%)	
TMJ acoustic symptoms	no	127 (90.7%)	111 (69.4%)	*p* = 0.000
	yes	11 (7.9%)	39 (24.4%)	
	I don’t know	2 (1.4%)	10 (6.3%)	
TMJ blocking	no	138 (98.6%)	147 (91.9%)	*p* = 0.028
	yes	2 (1.4%)	12 (7.5%)	
	I don’t know	0 (0%)	1 (0.6%)	
Teeth clenching	no	119 (85%)	87 (54.4%)	*p* = 0.000
	yes	18 (12.9%)	57 (35.6%)	
	I don’t know	3 (2.1%)	16 (10%)	
Teeth grinding	no	127 (90.7%)	109 (68.1%)	*p* = 0.000
	yes	8 (5.7%)	26 (16.3%)	
	I don’t know	5 (3.6%)	25 (15.6%)	
Increased masticatory muscles tension	no	125 (89.3%)	106 (66.3%)	*p* = 0.000
	yes	11 (7.9%)	34 (21.3%)	
	I don’t know	4 (2.9%)	20 (12.5%)	

**Table 3:** Comparison of type D personality components (NA and SI) in group P2 in relation to gender.

Variable	Female		Male		Statistical Significance
	$\bar{x}$	SD	$\bar{x}$	SD	
NA	11.73	6.00	9.25	6.06	*p* = 0.00
SI	10.09	4.98	8.75	5.32	*p* = 0.03

## Data Availability

The data presented in this study are available on request from the corresponding author. The data are not publicly available due to sensitive information.
